# Candidemia Following Ureteric Stent Placement in a Patient With Type 2 Diabetes Treated With Canagliflozin

**DOI:** 10.3389/fendo.2019.00020

**Published:** 2019-01-30

**Authors:** Rishi Raj, Jon Hendrie, Aasems Jacob, Derick Adams

**Affiliations:** ^1^Department of Endocrinology, Diabetes and Metabolism, University of Kentucky, Lexington, KY, United States; ^2^Department of Medicine, University of Kentucky, Lexington, KY, United States; ^3^Department of Hematology and Oncology, University of Kentucky, Lexington, KY, United States

**Keywords:** candidemia, candiduria, sodium-glucose co-transporter 2 inhibitors, canagliflozin, diabetes mellitus, ureteral stent

## Abstract

A 38-year-old female patient with well-controlled type 2 diabetes mellitus treated with canagliflozin underwent ureteral stent placement for obstructive renal calculi. Ten days following ureteroscopy and ureteral stenting, she developed fevers and blood cultures grew Candida glabrata (*C. glabrata*). The patient was successfully treated with an extended course of broad-spectrum antibiotics and antifungal agents. The clinical presentation of candidemia is indistinguishable from bacteremia resulting in delay in diagnosis and treatment. Candiduria is commonly seen in patients with type 2 diabetes, however it rarely leads to candidemia in an otherwise healthy person following a relatively simple urologic procedure. Sodium-glucose co-transporter 2 (SGLT-2) inhibitors act by its glycosuric effect and further increases the risk of genitourinary candida infection. Urologic procedures may lead to bloodstream entry of the genitourinary fungal organisms and result in life-threatening fungemia. Our case emphasizes the importance of awareness of the increased risk of potentially life threatening fungemia in patients using SGLT-2 inhibitors to avoid delay in diagnosis and treatment.

## Background

According to the most recent Centers for Disease Control and Prevention (CDC) data, ~46,000 healthcare-associated candida infections occur in the United States each year (1). Invasive Candida infections can be fatal, with a 30-day all-cause mortality of 30% and high rates of morbidity (2). Although, there are at least 15 distinct Candida species, more than 90% of the invasive diseases are caused by the five most common pathogens, *C. albicans, C. glabrata, C. tropicalis, C. parapsilosis*, and *C. krusei* (3). SGLT-2 inhibitors are known to increase the incidence of urogenital infections however, there have been no reported cases of invasive candidemia associated with SGLT-2 inhibitor use. Although these agents can potentiate the urinary colonization of candida, it is unknown whether this colonization is a preceding factor for the development of candidemia following urologic procedures. With our literature review, we propose that patient with type 2 diabetes treated with canagliflozin could be at a higher risk of candidemia following urological procedures like ureteral stent placement by bloodstream entry of the organism.

## Case Presentation

A 38-year-old Caucasian female presented to the emergency department with 3 days of left lower quadrant abdominal pain rated 6/10 with radiation to the left lower back. She also reported a high-grade fever (103°F) with associated nausea and vomiting. She denied dysuria or hematuria. Medical history was significant for well-controlled type II diabetes mellitus, hypertension, and hyperlipidemia. She was taking canagliflozin 100 mg daily, lisinopril 20 mg daily, and atorvastatin 80 mg daily. She has been on canagliflozin (SGLT-2 inhibitor) for type 2 diabetes mellitus for 14 months prior to the current presentation. She denied any history of urinary tract infections or renal stones. On presentation, her blood pressure was 172/86 mmHg, heart rate 94 beats per minute and temperature 102°F. BMI was 46.61 kg/m^2^. Physical examination revealed tenderness to palpation in the left lower quadrant and left flank. The rest of the examination was unremarkable. Urinalysis (Table 1) revealed pyuria, bacteriuria, and nitrites. Hemoglobin A1C was 7.5% (59 mmol/mol). CT abdomen and pelvis without contrast showed an obstructive 4–5 mm left distal ureteral stone associated with mild hydroureteronephrosis. She was diagnosed with obstructing nephrolithiasis complicated by pyelonephritis and was empirically treated with intravenous ceftriaxone 1 gram every 24 h. Cystoscopy with retrograde pyelography was done and a left 6-French × 24 cm double-J ureteral stent was placed. Placement was confirmed with fluoroscopy and cystoscopy. Intraoperative urine cultures obtained from the left renal pelvis and bladder showed no growth. She was discharged home on cefdinir 300 mg twice a day for 14 days and tamsulosin 0.4 mg daily for 30 days with a urology follow-up appointment in 2 weeks.

**Table 1:** Laboratory studies.

**Test (Units)**	**References range**	**1ST admission**	**2ND admission**
WBC count (k/uL)	3.7–10.3	7.4	12.1
Hemoglobin (g/dL)	11.2–15.7	13.8	13.6
Glucose level (mg/dL)	74-99	286	161
Urea nitrogen, blood (mg/dL)	7–21	9	9
Creatinine level (mg/dL)	0.60–1.10	0.69	0.60
Alanine aminotransferase (U/L)	8–33	43	41
Aspartate aminotransferase (U/L)	11–32	46	28
Alkaline phosphatase (U/L)	35–104	135	113
**URINALYSIS WITH MICROSCOPY**			
Specific gravity	1.001–1.030	≥1.030	1.025
pH	4.5–8.0	5.0	5.0
Protein (mg/dL)	–	30	≥300
Glucose (mg/dL)	–	≥1,000	Negative
Ketone	–	Trace	Trace
Blood	–	Small	Large
Nitrite	–	Positive	Positive
Leukocyte esterase	–	Negative	Small
WBC (/HPF)	0–5	16–30	>50
RBC (/HPF)	0–3	3	>50
Bacteria	–	Present	Present
Culture	–	10,000–100,000 CFU/ml mixed urogenital flora	>100,000 CFU/ml Klebsiella pneumoniae

Ten days later, she presented again with intermittent low-grade fever (100.5°F) and left flank pain. She was hypotensive (96/58 mmHg), tachycardic (104 bpm) and febrile with temperature of 102.8°F. Physical examination revealed severe tenderness to palpation in the left lower quadrant extending to the suprapubic area. Laboratory workup (Table 1) showed worsening leukocytosis, pyuria, bacteriuria, positive urinary nitrites, and small leukocyte esterase. A repeat CT scan of the abdomen and pelvis with renal stone protocol confirmed the position of the left ureteral stent and absence of previously seen stone. She was diagnosed with sepsis secondary to acute pyelonephritis in the setting of recent stent placement and started on aggressive intravenous hydration and Piperacillin-Tazobactam. Urine culture from the day of admission grew *Klebsiella pneumoniae* with >100 k CFU/ml however blood cultures remained negative. Despite appropriate antibiotics, she remained febrile even after 48 h. Two days later, the anaerobic blood cultures became positive for *C. glabrata*. She was started on intravenous micafungin 100 mg every 24 h and antibiotic was narrowed down based on culture sensitivities. Repeat cystoureteroscopy with retrograde pyelogram revealed partial contrast uptake in the left kidney due to an impacted ureteral stone in the mid ureter. The presence of fungal elements and cloudy urine were also found in the left kidney. The previously placed left ureteral stent was removed and a new double-J ureteral stent was placed. Urine from the left renal pelvis and bladder were cultured for a second time which grew *C. glabrata* susceptible to Fluconazole. On the 4th day of admission, blood cultures from both anaerobic and aerobic samples grew *C. glabrata*. Dilated fundus examination was negative for fungal ocular involvement and transthoracic echocardiogram was unremarkable for valvular vegetations. Blood cultures after antifungal initiation remained negative. She was eventually discharged on oral fluconazole 800 mg daily for 2 weeks and cefazolin 2 g every 8 h for 7 days.

## Discussion and Review of Literature

Canagliflozin, the first sodium-glucose co-transporter 2 (SGLT-2) inhibitor introduced in the USA was FDA approved in March 2013. Since then, SGLT-2 inhibitors have become popular in the management of type 2 diabetes mellitus as monotherapy (in the event of metformin intolerance), dual, or triple therapy. Although, it increases the incidence of urogenital infections, there is no report of candidemia associated with its use. We hereby discuss possible association of candiduria leading to candidemia following urologic procedure in a patient with diabetes on SGLT-2 inhibitors with current evidence from literature.

Candiduria is frequently encountered among patients with diabetes and is even higher in presence of certain underlying risk factors like female gender, indwelling urinary catheter, prior use of antibiotics, and acidic urine. However, it rarely results in candidemia. Ang et al. analyzed 249 cases of candidemia and reported 26 cases of candidemia originating from a urinary source in a retrospective review. However, most of the patients who developed candidemia from urinary source had some underlying risk factors like urinary tract abnormalities (88%), use of antibiotics within 1 week (85%), underlying malignancies (73%), urinary tract obstruction (73%), and prior urologic procedure within 2 weeks (73%), which put them at an increased risk of fungemia (4). Other similar studies also have not found any direct evidence of association between candiduria leading to candidemia without any of the underlying high risk factors (5–9). Candiduria, however is linked with increased mortality and is therefore considered as an independent marker of higher mortality (8, 10, 11). In a randomized control trial, Binelli et al. however found that candida isolates from urine and blood were different in 52% of patients with simultaneous candiduria and candidemia implying alternate source (12). Patients with diabetes are inherently at increased risk of candiduria due to glycosuria, immune dysfunction associated with hyperglycemia, and increased virulence of Candida (13, 14). Various studies have found candida to be third or fourth most commonly isolated organisms from the urine among hospitalized patients (7, 15, 16). A cross-sectional study on 305 patient with type 2 diabetes in an ambulatory setting showed presence of candiduria in 12.5% of patients, majority (95%) being in female (17). Nosocomial candiduria has even a higher prevalence ranging from 10 to 89% (7). Apart from uncontrolled diabetes, female gender, old age, presence of indwelling urinary catheter, prior use of antibiotics, and acidic urine pH are other known risk factors for urinary candida colonization (6, 7, 16–21).

Urologic procedure like ureteral stent placement may lead to bacterial (68–90%) and candidal (10–40%) colonization of urinary tract and is attributed to formation of a biofilm (22–25). Dariane et al. found candiduria to be frequently associated with ureteral stent placement, most commonly due to *C. albicans* (19–72%) followed by *C. glabrata* (15.6–49.4%) (25, 26). Two cases of candidemia was reported in elderly patients following ureteric stent placement for nephrolithiasis. Both patients had a positive candidal urine cultures at the time of procedure, prior broad-spectrum antibiotic use, and one of them was diabetic (27). Two cases of candidemia following ureteroscopy with stone manipulation was reported in patients with cirrhosis who underwent ureteric stent placement for obstructing stone and developed candidemia at the time of stent removal at 60 and 35 days (28). The mechanism of candidemia following ureteroscopy or ureteral stenting is proposed to be direct bloodstream seeding through microscopic lacerations in the presence of increased intra-ureteral pressure during ureteroscopy resulting in pyelovenous and pyelolymphatic reflux (25).

Our patient developed candidemia within 2 weeks following ureteric stent placement while on SGLT-2 inhibitor, raising concern about association of SGLT-2 inhibitor with increased risk of candidemia following ureteric stent placement. SGLT-2 inhibitors result in glycosuria up to 70–120 g per day (29). This pharmacologically-induced glycosuria may be implicated in the increased urogenital candida colonization in patients on SGLT2 inhibitors compared to placebo (10–31 vs. 3–14%) (30, 31). A recent study on adverse drug reports (ADRs) with SGLT-2 inhibitors (dapagliflozin, canagliflozin, and empagliflozin) in Spain over a period of 3.5-year showed increased reporting of urogenital tract infections (32).

Although, candida colonization is mostly asymptomatic and does not require treatment, Infectious Diseases Society of America (IDSA) recommends performing urine culture and to treat any positive urine culture prior to urological procedure (3, 25). When indicated, oral fluconazole, 400 mg (6 mg/kg) daily, OR Amphotericin B deoxycholate, 0.3–0.6 mg/kg daily is considered to be drug of choice (3). However, the duration of antifungal therapy required to achieve sterilization of urine is not well-established and current practice varies from 48 h to 3 weeks before stent exchange (25). Furthermore, several days of antifungal agents may not be practically feasible especially in situations where patient might require acute urologic interventions, e.g., ureteral stent placement for stone or obstruction.

Our case highlights a rare and unusual adverse event of canagliflozin in patient with diabetes who underwent ureteric stent placement. With increasing use of SGLT-2 inhibitors, more patients will be at risk of urinary tract colonization with candida, which might result in increased incidences of candida colonization related complications (e.g., symptomatic candiduria or candidemia following urologic surgery). Large comparative studies are required to address the necessity of antifungal therapy and its duration in preventing candidemia in patient with type 2 diabetes on SGLT-2 inhibitors undergoing urologic procedures. We also recommend discontinuation of SGLT-2 inhibitors prior to non-emergent/elective urologic interventions in order to decrease the colonization by candida, but more studies are needed to establish the exact duration of drug discontinuation required to result in appropriate urinary tract decolonization and optimal time for reinitiation.

## Conclusion

In summary, candida colonization of ureteral stents is common and can rarely lead to subsequent candidemia following urologic procedures especially in presence of risk factors. Due to the risk of urogenital candida infections in patients with diabetes treated with SGLT-2 inhibitors, urine culture and treatment of urinary colonization is advised before performing any urological procedures.

## Patient Consent

Written informed consent was obtained from the participant for publication of this case report.

## Author Contributions

JH contributed in preparing the case presentation. RR and AJ prepared the discussion of the manuscript. DA reviewed the manuscript in detail.

### Conflict of Interest Statement

The authors declare that the research was conducted in the absence of any commercial or financial relationships that could be construed as a potential conflict of interest.
